# Supplementary material for: Unveiling the age and origin of biogenic aggregates produced by earthworm species with their NIRS fingerprint in a subalpine meadow of Central Pyrenees
Source: PLoS One. 2020 Aug 12;15(8):e0237115. doi: 10.1371/journal.pone.0237115 (PMC7423103; doi:10.1371/journal.pone.0237115)
Supplement: S1 Table — (DOCX) [file pone.0237115.s001.docx]

| **S1 Table.** | | | | | | | | |  | |  | |  | |  | |  | |
| --- | --- | --- | --- | --- | --- | --- | --- | --- | --- | --- | --- | --- | --- | --- | --- | --- | --- | --- |
|  | **Family** | **Soil core** | |  |  |  |  |  | |  | |  | |  | |  | |  |
| **Phyllum (Subphyllum), Class, Order** |  | | I | I | II | II | III | III | | IV | | IV | | V | | V | |  |
|  |  | | Density (N) | Biomass (B) | N | B | N | B | | N | | B | | N | | B | |  |
| **Nematoda, Adenophorea** | Mermithidae | | 5 | 0.025 | - | - | - | - | | 1 | | 0.002 | | 6 | | 0.005 | |  |
| **Arthropoda (Myriapoda), Chilopoda: Geophilomorpha** | - | | - | - | - | - | - | - | | - | | - | | 2 | | 0.004 | |  |
| **Arthropoda (Hexapoda), Insecta: Coleoptera** | Staphylinidae | | 1 | 0.004 | 1 | 0.000 | - | - | | - | | - | | - | | - | |  |
|  | Curculionidae | | 3L | 0.038 | 5L | 0.042 | 4L+ 1 hueco | 0.059 | | 25L | | 0.284 | | 2+5L | | 0.079 | |  |
|  | Elateridae | | 2L | 0.016 | - | - | 2L | 0.008 | | 6L | | 0.076 | | 4L | | 0.133 | |  |
|  | Coccinelidae | | 1 | 0.003 | - | - | - | - | | - | | - | | - | | - | |  |
|  | Scarabeidae | |  |  | - | - | - | - | | 1 | | 0.010 | | - | | - | |  |
| **Arthropoda (Hexapoda), Insecta: Hymenoptera** | Formicidae | | 2 | 0.003 | 59+5L | 0.118 | 3 | 0.004 | | 2 | | 0.003 | | 34 | | 0.066 | |  |
| **Arthropoda (Hexapoda), Insecta: Diptera** | Unknown | | - | - | - | - | - | - | | - | | - | | 1+1L | | 0.010 | |  |
| **Arthropoda (Chelicerata), Arachnida: Araneae** | Liniphidae | | - | - | 1 | 0.001 | - | - | | - | | - | | 1 | | 0.001 | |  |
| **Arthropoda (Chelicerata), Arachnida: Opilionida** | Phalangiidae | | - | - | - | - | 1 (with 5 Acari stuck in the legs) | 0.028 | | - | | - | | - | | - | |  |
| **Mollusca, Gastropoda: Pulmonata** | Unknown | | 1 | 0.004 | 8 | 0.536 | 1 | 0.122 | | - | | - | | 1 | | 0.005 | |  |
| **Not determined** | - | | 1 | 0.001 | 3 | 0.014 | - | - | | 1 | | 0.00063 | | 3 | | 0.002 | |  |
|  | TOTAL (per core) | | 16 | 0.093 | 84 | 0.710 | 13 | 0.221 | | 38 | | 0.376 | | 62 | | 0.305 | |  |
|  | TOTAL (ind.m^-2^) | | 256 | 1.488 | 1344 | 11.366 | 208 | 3.530 | | 608 | | 6.009 | | 992 | | 4.887 | |  |
