# Supplementary material for: Unveiling the age and origin of biogenic aggregates produced by earthworm species with their NIRS fingerprint in a subalpine meadow of Central Pyrenees
Source: PLoS One. 2020 Aug 12;15(8):e0237115. doi: 10.1371/journal.pone.0237115 (PMC7423103; doi:10.1371/journal.pone.0237115)
Supplement: S2 Table — Analysis was performed with short wave NIR spectra (700 to 1,100 nm) after Savitzky-Golay 1st derivative transformation with 21 smoothing points (10, 10). (DOCX) [file pone.0237115.s002.docx]

**S2 Table. Results of the calibration model performance for each species and the variables measured in incubated earthworm casts (all dates) as assessed by the coefficient of determination of calibration (R^2^_c_), the root mean square error of cross validation (RMSECV), the number of factors of the PLS model.** Analysis was performed with short wave NIR spectra (700 to 1,100 nm) after Savitzky-Golay 1^st^ derivative transformation with 21 smoothing points (10, 10).

| **Species** | **Variables** | **R^2^_c_** | **RMSEC** | **SEC** | **R^2^_cval_** | **RMSECV** | **SECV** | **Factors** |
| --- | --- | --- | --- | --- | --- | --- | --- | --- |
| ***A. rosea*** | C | 0.964 | 0.16 | 0.16 | 0.415 | 0.67 | 0.69 | 5 |
|  | N | 0.924 | 0.03 | 0.03 | 0.274 | 0.09 | 0.10 | 5 |
|  | $\mathrm{NH}_{4}^{+}$ | 0.996 | 0.14 | 0.15 | 0.764 | 1.17 | 1.21 | 7 |
|  | $\mathrm{NO}_{3}^{-}$ | 0.705 | 5.62 | 5.78 | 0.316 | 9.06 | 9.32 | 3 |
| ***L. friendi*** | C | 0.997 | 0.05 | 0.05 | 0.259 | 0.85 | 0.88 | 7 |
|  | N | 0.995 | 0.006 | 0.006 | 0.101 | 0.08 | 0.09 | 7 |
|  | C:N | 0.978 | 0.008 | 0.008 | 0.545 | 0.04 | 0.04 | 5 |
|  | $\mathrm{NH}_{4}^{+}$ | 0.934 | 0.46 | 0.47 | 0.440 | 1.43 | 1.47 | 4 |
|  | $\mathrm{NO}_{3}^{-}$ | 0.997 | 0.45 | 0.46 | 0.126 | 8.13 | 8.38 | 7 |
| ***P. pyrenaicus*** | C | 0.511 | 0.48 | 0.50 | 0.197 | 0.66 | 0.67 | 2 |
|  | N | 0.477 | 0.05 | 0.05 | 0.083 | 0.07 | 0.07 | 2 |
|  | C:N | 0.994 | 0.006 | 0.006 | 0.381 | 0.06 | 0.07 | 7 |
|  | $\mathrm{NH}_{4}^{+}$ | 0.912 | 0.81 | 0.83 | 0.656 | 1.69 | 1.74 | 4 |
|  | $\mathrm{NO}_{3}^{-}$ | 0.993 | 0.80 | 0.83 | 0.687 | 5.92 | 6.09 | 6 |
