# Supplementary material for: Unveiling the age and origin of biogenic aggregates produced by earthworm species with their NIRS fingerprint in a subalpine meadow of Central Pyrenees
Source: PLoS One. 2020 Aug 12;15(8):e0237115. doi: 10.1371/journal.pone.0237115 (PMC7423103; doi:10.1371/journal.pone.0237115)
Supplement: S3 Table — Analysis was performed with NIR spectra from 1,100 to 2,500 nm after Savitzky-Golay 2nd derivative transformation with 21 smoothing points (10, 10). (DOCX) [file pone.0237115.s003.docx]

**S3 Table. Results of the calibration model performance for each species and the variables measured in incubated earthworm casts (all dates) as assessed by the coefficient of determination of calibration (R^2^_c_), the root mean square error of cross validation (RMSECV), the number of factors of the PLS model.** Analysis was performed with NIR spectra from 1,100 to 2,500 nm after Savitzky-Golay 2^nd^ derivative transformation with 21 smoothing points (10, 10).

| **Species** | **Variables** | **R^2^_c_** | **RMSEC** | **SEC** | **R^2^_cval_** | **RMSECV** | **SECV** | **Factors** |
| --- | --- | --- | --- | --- | --- | --- | --- | --- |
| ***A. rosea*** | C | 0.998 | 0.03 | 0.03 | 0.426 | 0.67 | 0.69 | 6 |
|  | N | 0.554 | 0.07 | 0.07 | 0.229 | 0.10 | 0.10 | 1 |
|  | C:N | 0.516 | 0.07 | 0.07 | 0.083 | 0.10 | 0.11 | 1 |
|  | $\mathrm{NH}_{4}^{+}$ | 0.990 | 0.22 | 0.23 | 0.615 | 1.50 | 1.54 | 4 |
|  | $\mathrm{NO}_{3}^{-}$ | 0.765 | 5.01 | 5.16 | 0.101 | 10.4 | 10.68 | 2 |
| ***L. friendi*** | C | 0.652 | 0.55 | 0.56 | 0.059 | 0.96 | 0.99 | 1 |
|  | N | 0.601 | 0.05 | 0.05 | NA | 0.09 | 0.09 | 1 |
|  | C:N | 0.999 | 0.002 | 0.002 | 0.373 | 0.05 | 0.05 | 6 |
|  | $\mathrm{NH}_{4}^{+}$ | 0.972 | 0.30 | 0.31 | 0.289 | 1.61 | 1.66 | 3 |
|  | $\mathrm{NO}_{3}^{-}$ | 0.643 | 4.87 | 5.03 | NA | 8.85 | 9.14 | 1 |
| ***P. pyrenaicus*** | C | 0.998 | 0.03 | 0.03 | 0.449 | 0.54 | 0.56 | 6 |
|  | N | 0.996 | 0.004 | 0.004 | 0.371 | 0.06 | 0.06 | 6 |
|  | C:N | 0.890 | 0.02 | 0.03 | 0.203 | 0.07 | 0.07 | 3 |
|  | $\mathrm{NH}_{4}^{+}$ | 0.932 | 0.71 | 0.73 | 0.293 | 2.43 | 2.49 | 3 |
|  | $\mathrm{NO}_{3}^{-}$ | 0.976 | 1.54 | 1.59 | 0.268 | 9.05 | 9.31 | 4 |
