# Supplementary figures and images for: Unveiling the age and origin of biogenic aggregates produced by earthworm species with their NIRS fingerprint in a subalpine meadow of Central Pyrenees
Source: PLoS One. 2020 Aug 12;15(8):e0237115. doi: 10.1371/journal.pone.0237115 (PMC7423103; doi:10.1371/journal.pone.0237115)

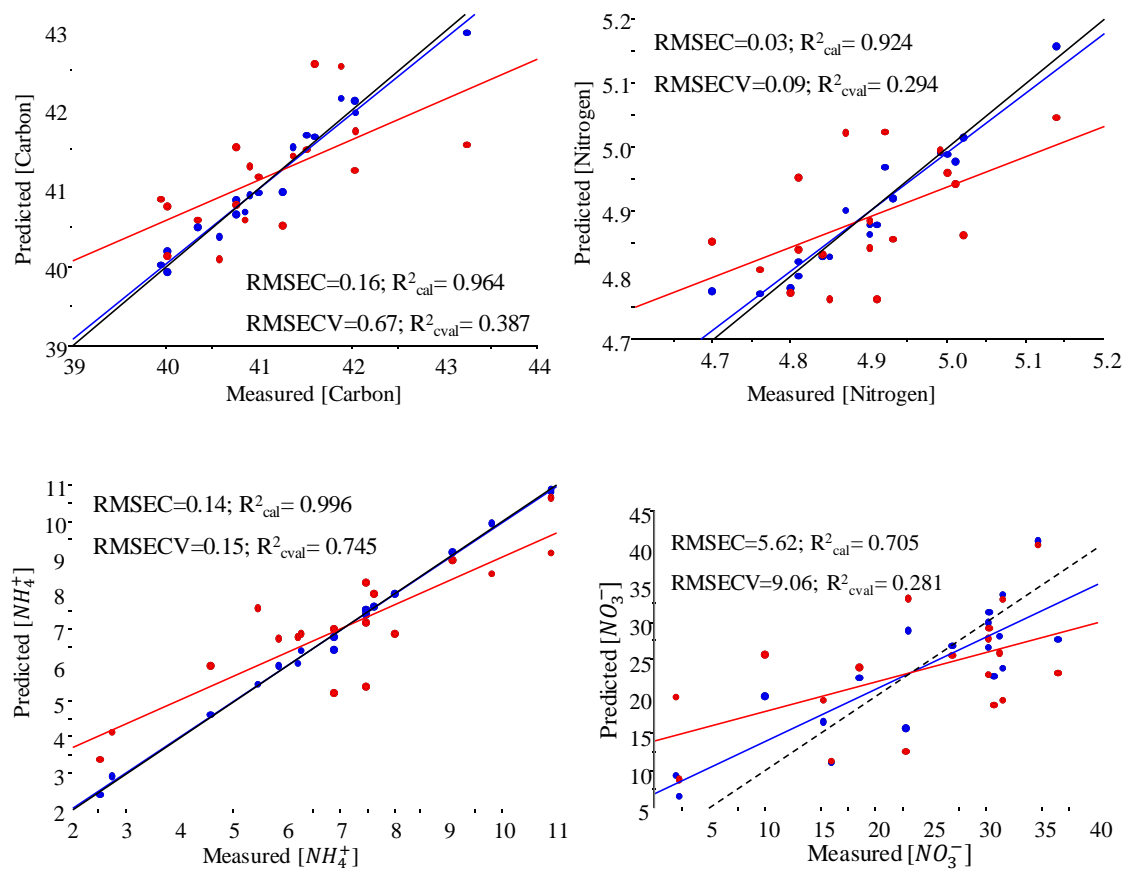

**S1 Fig.**

Supplement: S1 Fig — The C:N ratio is not shown (the correlation coefficient was very low). (PDF) [file pone.0237115.s004.pdf]

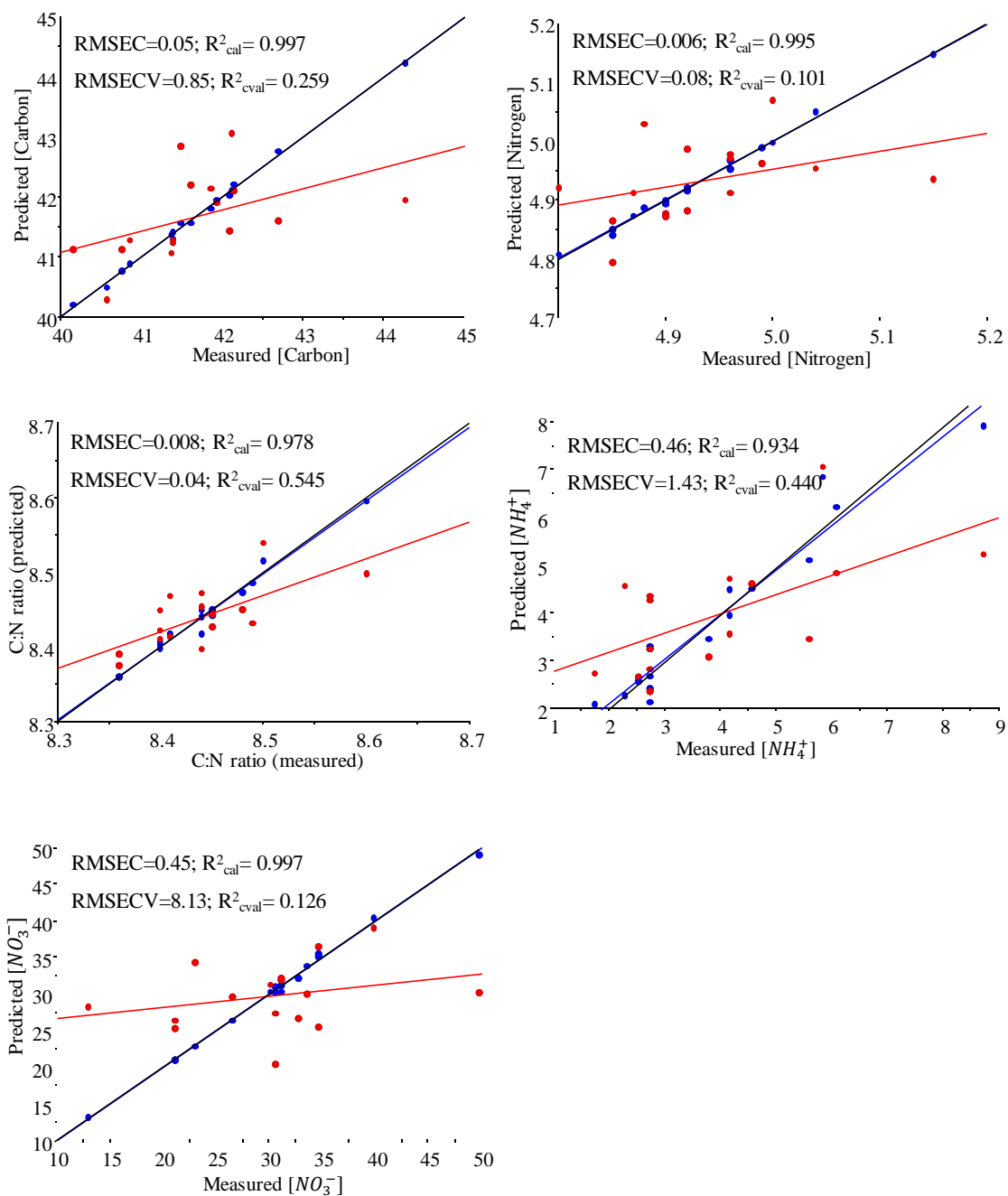

**S2 Fig.**

Supplement: S2 Fig — (PDF) [file pone.0237115.s005.pdf]

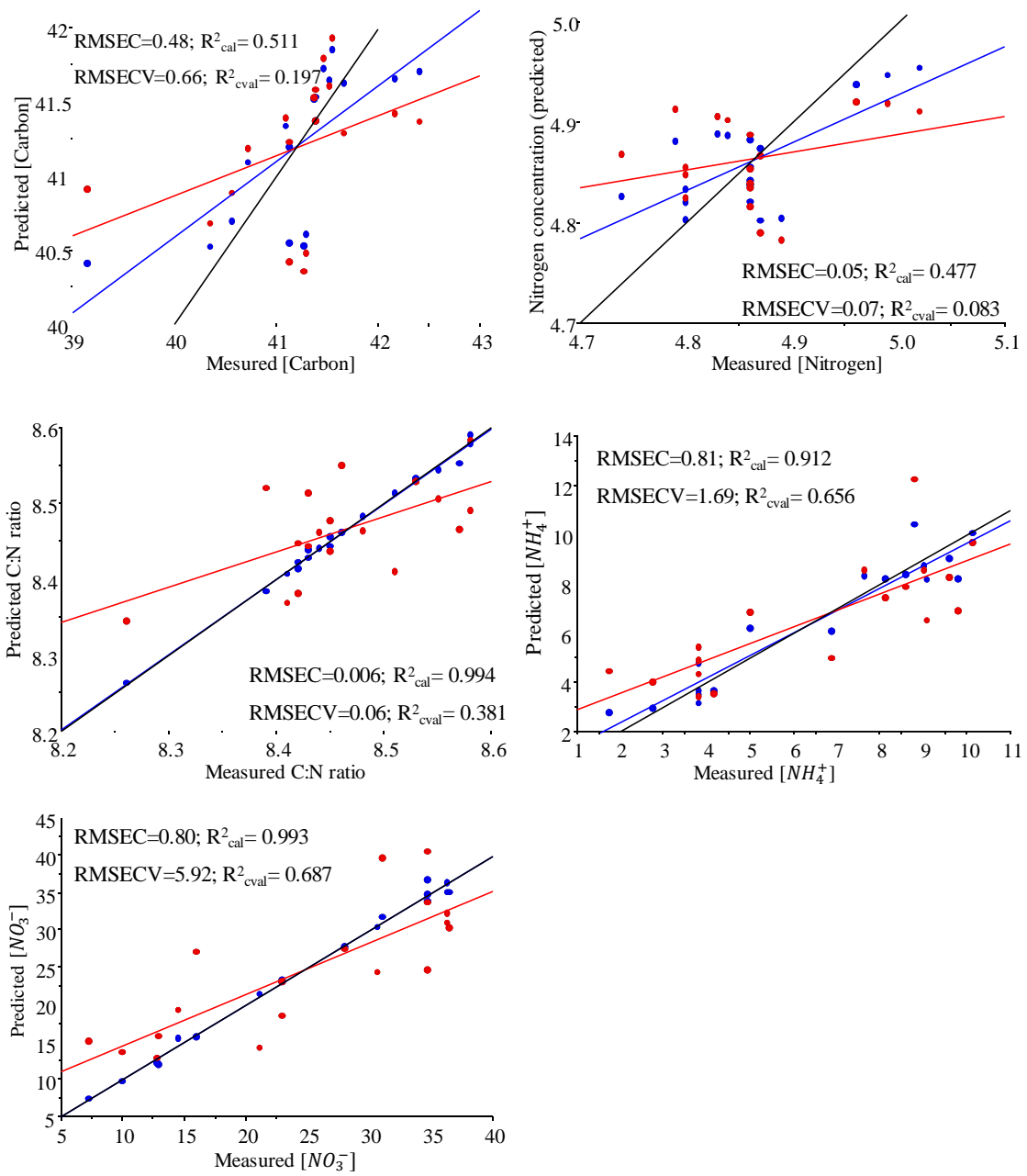

**S3 Fig.**

Supplement: S3 Fig — (PDF) [file pone.0237115.s006.pdf]

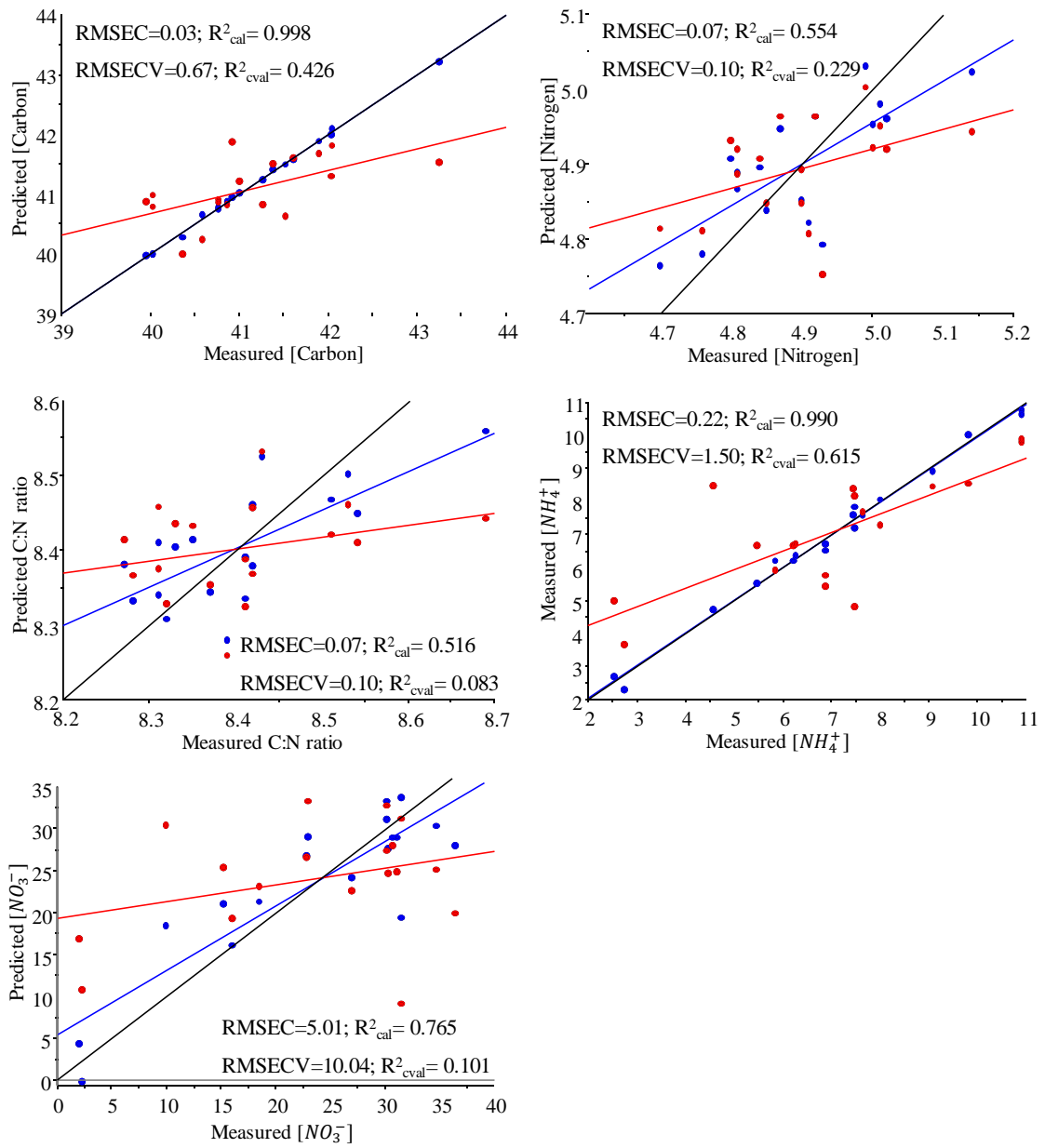

**S4 Fig.**

Supplement: S4 Fig — (PDF) [file pone.0237115.s007.pdf]

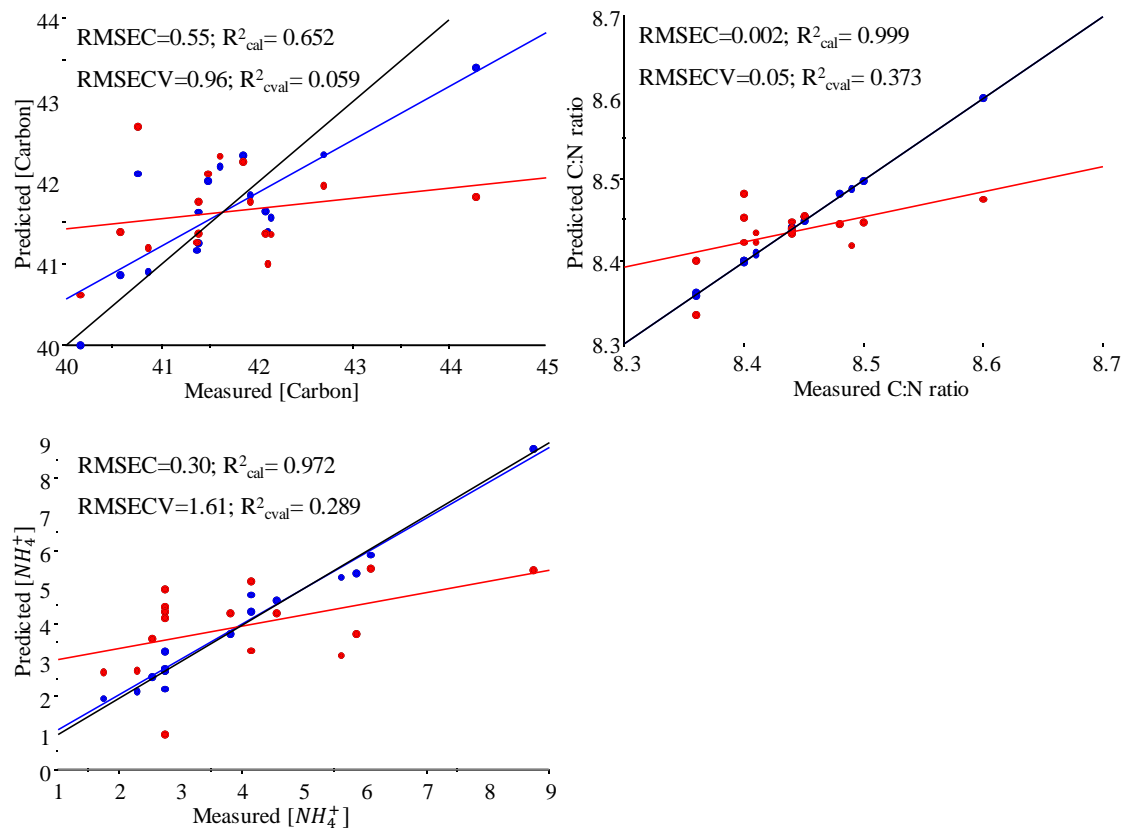

**S5 Fig.**

Supplement: S5 Fig — N and NO3 concentrations are not shown (very low coefficient of correlation). (PDF) [file pone.0237115.s008.pdf]

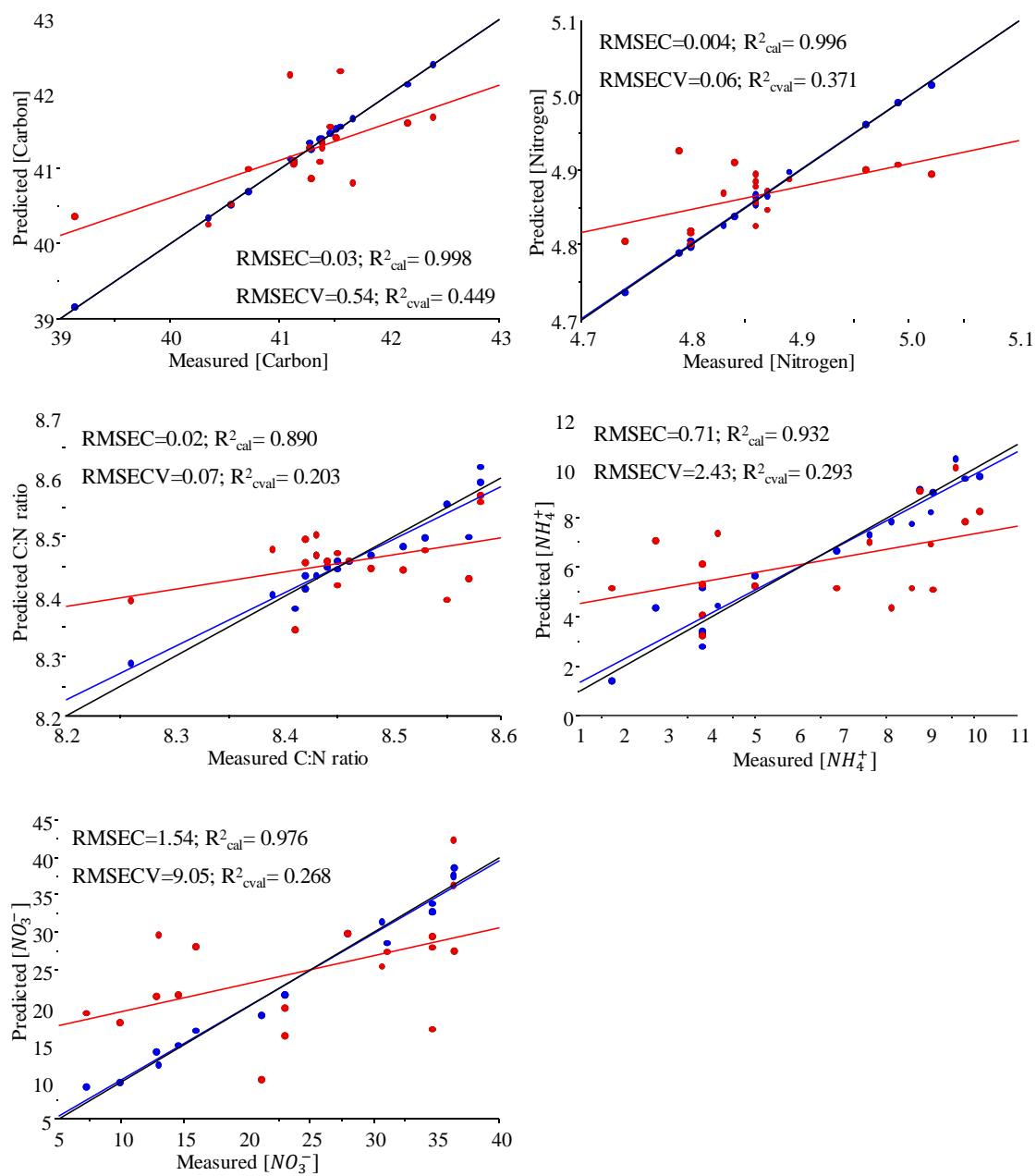

**S6 Fig.**

Supplement: S6 Fig — (PDF) [file pone.0237115.s009.pdf]
