# Supplementary material for: Unveiling the age and origin of biogenic aggregates produced by earthworm species with their NIRS fingerprint in a subalpine meadow of Central Pyrenees
Source: PLoS One. 2020 Aug 12;15(8):e0237115. doi: 10.1371/journal.pone.0237115 (PMC7423103; doi:10.1371/journal.pone.0237115)
Supplement: Attachment — Submitted filename: Response to Reviewers.docx [file pone.0237115.s010.docx]

**PONE-D-20-10139**
Unveiling the age and origin of biogenic aggregates produced by earthworm species with their NIRS fingerprint in a subalpine meadow of Central Pyrenees
PLOS ONE

Dear Dr Jimenez,

Thank you for submitting your manuscript to PLOS ONE. After careful consideration, we feel that it has merit but does not fully meet PLOS ONE’s publication criteria as it currently stands. Therefore, we invite you to submit a revised version of the manuscript that addresses the points raised during the review process.


We would appreciate receiving your revised manuscript by recent 8 weeks. When you are ready to submit your revision, log on to https://www.editorialmanager.com/pone/ and select the 'Submissions Needing Revision' folder to locate your manuscript file.

If you would like to make changes to your financial disclosure, please include your updated statement in your cover letter.

To enhance the reproducibility of your results, we recommend that if applicable you deposit your laboratory protocols in protocols.io, where a protocol can be assigned its own identifier (DOI) such that it can be cited independently in the future. For instructions see: <http://journals.plos.org/plosone/s/submission-guidelines#loc-laboratory-protocols>

Please include the following items when submitting your revised manuscript:

- A rebuttal letter that responds to each point raised by the academic editor and reviewer(s). This letter should be uploaded as separate file and labeled 'Response to Reviewers'. [OK]
- A marked-up copy of your manuscript that highlights changes made to the original version. This file should be uploaded as separate file and labeled 'Revised Manuscript with Track Changes'. [OK]
- An unmarked version of your revised paper without tracked changes. This file should be uploaded as separate file and labeled 'Manuscript'. [OK]

Please note while forming your response, if your article is accepted, you may have the opportunity to make the peer review history publicly available. The record will include editor decision letters (with reviews) and your responses to reviewer comments. If eligible, we will contact you to opt in or out.

We look forward to receiving your revised manuscript.

Kind regards,

Fuzhong Wu
Academic Editor
PLOS ONE

Additional Editor Comments:

One reviewer suggested to reject it, but I think a chance should be given to the authors because another reviewer showed positive comments. Anyway, it has highly possibility to decline it, if the authors have not addressed all of the comments.

[OK, we do really thank the Editor for this decision and the chance given to us to submit a revised version of this research]
Journal Requirements:

When submitting your revision, we need you to address these additional requirements.

1. Please ensure that your manuscript meets PLOS ONE's style requirements, including those for file naming. The PLOS ONE style templates can be found at

https://journals.plos.org/plosone/s/file?id=wjVg/PLOSOne_formatting_sample_main_body.pdf and

https://journals.plos.org/plosone/s/file?id=ba62/PLOSOne_formatting_sample_title_authors_affiliations.pdf

[OK, we initially followed the guidelines of formatting and style, but it seems something went wrong. We have gone through the style requirements again, hoping that now the requirements are met.]

2. In your Methods section, please provide additional information regarding the permits you obtained for the work. Please ensure you have included the full name of the authority that approved the field site access and, if no permits were required, a brief statement explaining why.

[OK, the site where the research was conducted is an area that has been studied by different researchers from IPE-CSIC during the last 60 years. These studies covered aspects of general ecology, botany, geomorphology and other scientific disciplines, ranging from pastoral use, plant vegetation composition and dynamics, snow dynamics, tree line succession and woody encroachment processes due to land abandonment. We are granted permission from the Government of Aragón, no paperwork required. We just need to ask permission from the mayor of the near village (Fanlo) to access the site through a restricted unpaved way. We have mentioned it in the acknowledgement section.]

3. During our internal checks, the in-house editorial staff noted that you conducted research or obtained samples in another country. Please check the relevant national regulations and laws applying to foreign researchers and state whether you obtained the required permits and approvals. Please address this in your ethics statement in both the manuscript and submission information.

[No, we have neither conducted research in another country, nor obtained samples outside Spain. The study was conducted in the Central Pyrenees, the mountainous region that limits the border between Spain and France, but all samples were collected on the Spanish side. We are attaching a map (Google Earth) to indicate the site area (see below). The yellow line sets the border between Spain and France.

4. Thank you for stating the following in the Acknowledgments Section of your manuscript:

'..The ARAID foundation is acknowledged for support to Juan J. Jiménez. We acknowledge support of the publication fee by the CSIC Open Access Publication Support Initiative through its Unit of Information Resources for Research (URICI).'

We note that you have provided funding information that is not currently declared in your Funding Statement. However, funding information should not appear in the Acknowledgments section or other areas of your manuscript. We will only publish funding information present in the Funding Statement section of the online submission form.

[OK, the sentence in the acknowledgement section “The ARAID foundation is acknowledged for support to Juan J. Jiménez” does not refer to any funding support, that is why it was not mentioned in the Funding Statement section of the online submission form. It is just that those researchers from ARAID must state that sentence in the acknowledgements section.

With regards to the CSIC Open Access Publication Support Initiative we also need to include it in the acknowledgement section, as they will cover part of the Article Processing Charges.]

Please remove any funding-related text from the manuscript and let us know how you would like to update your Funding Statement. Currently, your Funding Statement reads as follows:

'YDH was supported by a COLCIENCIAS grant, Colombia (Code: 1116-569-34827) for a stay at IPE-CSIC (Spain). EV, PL, AZ, SG did not receive specific funding. The funders had no role in study design, data collection and analysis, decision to publish, or preparation of the manuscript.'

[Yes, this is correct]

a. Please clarify the sources of funding (financial or material support) for your study. List the grants or organizations that supported your study, including funding received from your institution.
b. State what role the funders took in the study. If the funders had no role in your study, please state: “The funders had no role in study design, data collection and analysis, decision to publish, or preparation of the manuscript.”
c. If any authors received a salary from any of your funders, please state which authors and which funders.

[No salary was received]

d. If you did not receive any funding for this study, please state: “The authors received no specific funding for this work.”

[OK, we have stated this: “The authors received no specific funding for this work.”]

Please include your amended statements within your cover letter; we will change the online submission form on your behalf.

[OK, we have included all these statements in the rebuttal cover letter.]

5. Please include captions for your Supporting Information files at the end of your manuscript, and update any in-text citations to match accordingly. Please see our Supporting Information guidelines for more information: [http://journals.plos.org/plosone/s/supporting-information](about:blank)

[OK, captions for supporting Information files have been included at the end of the manuscript. The figures in the Supporting Information section have been saved in pdf format, independently.]

[Note: HTML markup is below. Please do not edit.]

Reviewers' comments:

Reviewer's Responses to Questions

**Comments to the Author**

1. Is the manuscript technically sound, and do the data support the conclusions?

The manuscript must describe a technically sound piece of scientific research with data that supports the conclusions. Experiments must have been conducted rigorously, with appropriate controls, replication, and sample sizes. The conclusions must be drawn appropriately based on the data presented.

Reviewer #1: Partly

Reviewer #2: Yes

2. Has the statistical analysis been performed appropriately and rigorously?

Reviewer #1: N/A

Reviewer #2: Yes

3. Have the authors made all data underlying the findings in their manuscript fully available?

The [PLOS Data policy](http://www.plosone.org/static/policies.action#sharing) requires authors to make all data underlying the findings described in their manuscript fully available without restriction, with rare exception (please refer to the Data Availability Statement in the manuscript PDF file). The data should be provided as part of the manuscript or its supporting information, or deposited to a public repository. For example, in addition to summary statistics, the data points behind means, medians and variance measures should be available. If there are restrictions on publicly sharing data—e.g. participant privacy or use of data from a third party—those must be specified.

Reviewer #1: Yes

Reviewer #2: Yes

4. Is the manuscript presented in an intelligible fashion and written in standard English?

PLOS ONE does not copyedit accepted manuscripts, so the language in submitted articles must be clear, correct, and unambiguous. Any typographical or grammatical errors should be corrected at revision, so please note any specific errors here.

Reviewer #1: Yes

Reviewer #2: Yes

5. Review Comments to the Author

Please use the space provided to explain your answers to the questions above. You may also include additional comments for the author, including concerns about dual publication, research ethics, or publication ethics. (Please upload your review as an attachment if it exceeds 20,000 characters)

Reviewer #1: My major concerns are:
First, the Introduction section was not written properly and can be improved by 1) giving the background information and importance of this topic to the reader; 2) identifying what are unknown and what are the questions; and 3) letting the reader know what you intend to do in order to answer these questions.

[OK, we have rewritten some sections of the introduction to add clarity, emphasizing what remains unknown, the hypothesis tested in this study and the questions pending to be answered. What we intended to do in order to answer these questions was explicitly stated in the methods section]
By the way, your hypothesis should come before your aims rather than the other way around.

[OK, we have include the hypothesis before the aims of the study.]
Second, the results section was too lengthy. I even found a mass of the description about the supplementary materials. However, compared to the abundant content, the discussion section was too shallow.

[OK, we thank this reviewer comment, but since the technique is quite specific we believed it should be explained at its maximal length to ease understanding of the readers and likely researchers willing to repeat this kind of experimentation. However, in section **Calibration model performance and PLSR**, tables 5 and 6 have been moved to supplementary material as S2 Table and S3 table.

The Discussion section has been readapted and a new heading was included: **Pros and cons of the intended NIR assessment approach in field conditions**. Now we do believe it is more adjusted to the length of the results section.]
Third, and most important, the experiment consists of multiple models, it is unclear how these (such as the lines 269-277, vary in the “n” in the table 4) has been incorporated in the statistical models. Specifically, the post-hoc tests are not described and explained in the Methods section. Together, this makes it impossible to assess the robustness of your models, and hence, the robustness of your conclusions.

[OK, regarding data of table 4 and the use of posthoc comparisons as indicated by the reviewer #1, the analysis of variance showed that there were no significant differences in the concentration of the three elements analysed (C, N and C:N), so there is no reason to include in the table the post-hoc comparisons. We have reworded the sentence. We do hope it is clearer now.

Moreover, there are several statistical analyses in this study but this one specifically was analysed with ANOVA, and no subsequent analyses were needed. For the rest of statistical procedures, as PCA and PLS regression, these are explained in the methods section.]

Accordingly, although the key findings of the paper are of potential interest to PLoS ONE, there are some problems still need to be addressed before it’s publication. After revision, a re-submitted version may be further considered.

[OK, we do really thank the reviewer for his/her comments and for the opportunity given to resubmit a revised version of the manuscript.]

Minor comments:
Line31: Please check the usage of parentheses for the first and second species.

[OK, only the authorship for the first species was within parentheses]
The purposes, as well as the results, in the abstract section was too separated, such as the lines 38-41 and lines 45-47.

[OK, the sentence between lines 45-47 was moved immediately after the sentence in lines 38-41]

Line101: Please make sure that the soils all be named using WRB classification.

[OK, soils have been named using WRB classification, and the reference has been updated]

Line109-111: Repeat information: “dominated by…” and “as dominant plant species”

[OK, the word dominated has been replaced by characterized and “as dominant plant species” has been kept]

Line113: see my comments on line31. In addition, the abbreviations should be clear, you may consider to write the full names in the first mentioning in the main text.

[OK, the usage of parentheses has been checked and acronym was used after first mentioning the words organic matter]
The language needs to be improved by a native speaker before re-submission. For example, Lines129-130, and other places throughout the manuscript.

[OK, the language was checked by a native English speaking person. The term at a naked eye is quite common in our discipline when describing how different structures and soil organisms found in the soil sample are hand-sorted. However we have removed the term at the naked eye.]

Line257-260: According to the Table 3, I can’t address the meaning the author wants to presented. Is there something error? maybe I am mistaken, but should be clarified.

[OK, we have rewritten this paragraph. The protocol of collecting the items in the 9 soil cores was described previously in the materials and methods section.

Now the sentence reads: “The biomass of aggregates attached to roots and non-macroaggregated soil was 122.3 and 134.8 g m^-2^, respectively, while biomass of free (particulate) organic matter and invertebrate biogenic aggregates was 62.9 and 41.7 g m^-2^, respectively (Table 3)”.]

Lines264-266: I couldn’t find the relevant information in the table 3 at all.

[The figures indicated in those lines refers to an estimation of the biomass in terms of the surface area occupied by the soil cores; each soil core had a surface area of 10×10 cm2, if we extrapolate the value of 41.7 g in 10 cm^2^ to Kg m^-2^ we obtain 41.7 Kg. We have rephrased this sentence to improve clarity].

Lines273-274: see my major comments.

[OK, regarding data of table 4 and the use of posthoc comparisons as indicated by the reviewer #1, the analysis of variance showed that there were no significant differences in the concentration of the three elements analysed (C, N and C:N), so there is no reason to include in the table the post-hoc comparisons. We have reworded the sentence. We do hope it is clearer now].

Lines323-330: If the relevant information is valuable. Why you put them in the supplementary material.

[OK, yes, thanks for this comment; we had actually included this information in the main text before submission but later was moved to supplementary information section. We have put back the figure (new figure 4) that was in the Supporting Information as S1 a-f in the original submission.]

Lines471-472, lines 496-497 and lines 522-523: Please rephrase.
[OK, the sentences have been rephrased:

Lines 471-472: Comparison of our results with those obtained in other studies is not possible as the approach used in this study, i.e., linking field aggregates of the soil matrix, not only fresh casts, to NIR signals was not assessed in previous works.

Lines 496-497: These authors collected fresh casts from the field to compare and match their NIR signals with the casts produced by the same species in the lab. Our study, on the contrary, is the first one aiming at pairing the NIRS spectra fingerprints of ageing biogenic aggregates with the respective species that likely produced those aggregates.

Lines 522-523: *Octolasion lacteum* and *O. cyaneum* were not found at the time of sampling in the field, so no casts were available for both species.]

Reviewer #2: Review Domínguez-Haydar et al: Unveiling the age and origin of biogenic aggregates produced by earthworm species with their NIRS fingerprint in a subalpine meadow of Central Pyrenees

The manuscript PONE-20-10139 by Domínguez-Haydar et al presents a very interesting study aiming to acquire data on the production rate and spatio-temporal dynamics of earthworm induced macroaggregation in mountain soils and explore the capacity of NIRS to discriminate the age and origin (species) of a given soil macroaggregate (ped) under field conditions in subalpine pastures.
By combining the NIR spectral signatures of biogenic aggregates, root-aggregates, and non-aggregated soil and soil carbon (C), nitrogen (N), 4+ and 3− data, the authors suggest that a NIRS biogenic background noise (BBN) is present in the soil as a result of earthworm activity.
Overall, I think the topic of the manuscript is interesting and experiment design is rather reasonable. The results provide insights on how to analyse the role of soil fauna (e.g. earthworm) in important ecological processes of soil macroaggregation and associated organic matter dynamics by means of analyzing the BBN in the soil matrix.

However, before accepting to publication some minor revisions should be considered.

First, the results section is very detail. However, I feel that the results section is currently not well structured and the main results of the study are easy to miss in the large number of index, graphs, tables and analysis. I would recommend for the authors to thoroughly revise this section focusing more on the novel core results.

[OK, we have tried to counterbalance the comments of both reviewers, as Reviewer 1 suggested to include one more graph in the main text. We have moved some of the tables from this section to supplementary material section and focused more on the main aspects of the analysis to explore the capacity of NIRS to discriminate the age and origin (species) of a given soil macroaggregate and emphasize the novel core results.

We also believe that it is necessary to show with detail the sequence of steps in the analysis of the NIR spectra.]

Second, the structure of the manuscript and tables should follow the guideline of the PONE.

6. PLOS authors have the option to publish the peer review history of their article ([what does this mean?](https://journals.plos.org/plosone/s/editorial-and-peer-review-process#loc-peer-review-history)). If published, this will include your full peer review and any attached files.
[OK, we agree to the option to publish the peer review history of this paper if finally accepted].

If you choose “no”, your identity will remain anonymous but your review may still be made public.


**Do you want your identity to be public for this peer review?** For information about this choice, including consent withdrawal, please see our [Privacy Policy](https://www.plos.org/privacy-policy).

Reviewer #1: No

Reviewer #2: No

[NOTE: If reviewer comments were submitted as an attachment file, they will be attached to this email and accessible via the submission site. Please log into your account, locate the manuscript record, and check for the action link "View Attachments". If this link does not appear, there are no attachment files to be viewed.]

While revising your submission, please upload your figure files to the Preflight Analysis and Conversion Engine (PACE) digital diagnostic tool, <https://pacev2.apexcovantage.com/>. PACE helps ensure that figures meet PLOS requirements. To use PACE, you must first register as a user. Registration is free. Then, login and navigate to the UPLOAD tab, where you will find detailed instructions on how to use the tool. If you encounter any issues or have any questions when using PACE, please email us at [figures@plos.org](mailto:figures@plos.org). Please note that Supporting Information files do not need this step.
