# Supplementary material for: Unveiling the age and origin of biogenic aggregates produced by earthworm species with their NIRS fingerprint in a subalpine meadow of Central Pyrenees
Source: PLoS One. 2020 Aug 12;15(8):e0237115. doi: 10.1371/journal.pone.0237115 (PMC7423103; doi:10.1371/journal.pone.0237115)
Supplement: Attachment — Submitted filename: Response to Reviewers.docx [file pone.0237115.s011.docx]

**PONE-D-20-10139R1**
Unveiling the age and origin of biogenic aggregates produced by earthworm species with their NIRS fingerprint in a subalpine meadow of Central Pyrenees
PLOS ONE

6. Review Comments to the Author

Please use the space provided to explain your answers to the questions above. You may also include additional comments for the author, including concerns about dual publication, research ethics, or publication ethics. (Please upload your review as an attachment if it exceeds 20,000 characters)

Reviewer #1: Abstract：
Redundant information: produced in the lab. produced in the lab [OK, corrected]
In the abstract: provide more quantitative data. [OK, more quantitative data have been provided in the abstract]

Introduction：
The results will be useful for unveiling the contribution of earthworms in soil aggregation and soil organic matter (SOM) dynamics, so that this methodology can be used in other studies with other earthworm species. Rewrite the objective. [OK, the objective has been rewritten; we have changed this paragraph a bit. The main objective was to unveil the contribution of earthworms in soil aggregation and soil organic matter (SOM) dynamics in mountain soils.

The last sentence in the introduction emphasizes the need for replicated studies with other earthworm communities and/or soil invertebrates producing biogenic structures by using the same NIRS and PLS regression methodology.]

Results：
“is listed in The concentrations of C, N and C:N ratio in the three types of soil aggregates identified (Table 4)” rewrite. [OK, corrected. “The concentrations of C, N and C:N ratio in the three types of soil aggregates identified (Table 4) did not differ statistically (ANOVA, p>0.05)]
Rewrite the following words or sentence: If we; We also analysed. These phrases are useless, start with the results and conclusions directly. [OK, the sentences have been rewritten. “When these data were extrapolated to kg per meter square, the activity of …..”]

Discussion：
Several factors affected the variability of NIR spectral signals that were not addressed in this study:
1) Abiotic, such as water flow and dry-rewetting cycles that may disrupt aggregates, and
2) Biotic, like roots morphology, microbial biomass, and particulate organic matter in the soil.
3) human-induced, related to the process of identifying and separating BA from the soil block that may result in less precise selection.
- Delete this “conclusion”, this means that your study does not have any additional value for the research topic. [OK, these factors were not tested in this study. We have removed all factors. We have however included a sentence about the importance of expertise of students, researchers, etc. in the identification and selection of biogenic aggregates from field samples.]

Reviewer #2: This paper is much improved after revision and it meets the standards of journal publication. I would like to "Accept"

[OK, we are very grateful to the reviewer for the acceptance of this paper]
